# Grape Molasses as a Healthy Natural Ingredient in Hazelnut Butter: Effects on Emulsion Stability, Physicochemical, Nutritional, and Sensory Characteristics

**DOI:** 10.1021/acsomega.5c05563

**Published:** 2025-08-13

**Authors:** Soykan Gültekin, Emre Turan, Atilla Şimşek

**Affiliations:** Department of Food Engineering, Faculty of Agriculture, 220076 Ordu University, Ordu 52200, Türkiye

## Abstract

This study investigated the emulsion stability, physicochemical properties, nutritional content, and sensory characteristics of hazelnut butter formulations (*GMHB*) enriched with grape molasses (*GM*) as alternative to starch-based refined sugar (*SBRS*) or beet sugar both with and without palm oil stabilizer (*Stb*) and skimmed milk powder (*SMP*). The incorporation of *GM* increased the Hunter L*, a*, and b* values, while *Stb* and *SMP* generally reduced color values and increased %*FFA* (*P* < 0.05). The viscosity of *GMHB* formulations increased with higher *GM* and *Stb* ratios. Group-A samples (14.5–15% GM) exhibited higher total phenolic content (*TPC*) than Group-B (24–25% *GM*), with the highest *TPC* and viscosity found in samples A1 and B1 containing 3% *Stb* (*P* < 0.05). Total tocopherol content peaked in sample A1 (*P* < 0.05). *DPPH* antiradical activity and *PV* values were similar in all samples (*P* > 0.05). Increasing *GM* ratios enhanced the average levels of K (5.2%), Na (10%), and Ca (11%) but decreased some microelements. In Accelerated oil separation (*AOS*) and cumulative oil separation (*COS*) were raised in B-Group samples with high GM, regardless of *Stb* and *SMP*, with the least separation noted in A1, respectively, 8.63% and 0.23%. Mathematical equations (*R* ^2^ = 99.68%) relating *COS* and storage can predict emulsion stability in *GMHB* samples. Samples A1 and B1 with 3% *Stb* had the longest shelf life at both storage temperatures. Samples A2 and A3 received higher sensory evaluation scores. *GMHB* has functional potential due to its enhanced nutritional profile, which includes antioxidants like colorants, phenolics, and fatty acids, as well as minerals, dietary fiber, and vitamins.

## Graphical abstract

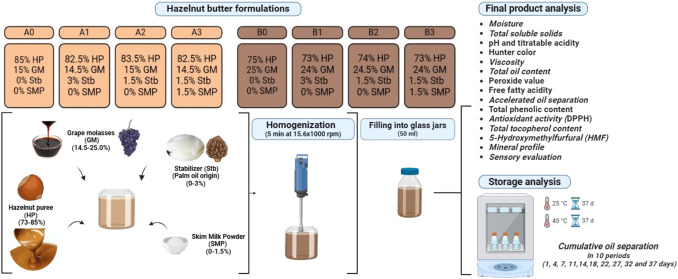

## 1 Introduction

People’s desire for a healthy and quality life has changed their diet. Nowadays, people have turned to foods that protect and improve their health during nutrition and reduce the risk of disease, protect health and improve well-being. Therefore, foods that can fulfill these demands, are consumed with the daily diet, are nutritious and do not contain synthetic compounds are called functional foods.^1^

Hazelnut (*Corylus avellana* L.) is considered as a functional food due to its significant contribution to human health and nutrition. Hazelnut contains 10–24% protein, 50–73% fat and 10–22% carbohydrate. It is rich in oleic fatty acid (84%), followed by linoleic (18.5%), palmitic (6%), stearic (3%) and linolenic (0.9%) fatty acids. 100 g hazelnut contains Vit E (36.6 mg), B1 (0.46 mg), B2 (0.13 mg), B6 (0.7 mg), niacin (2.1 mg), pantothenic acid (1.19 mg) and Vit A (69 IU) as well as amino acids such as arginine, histidine, isoleucine, leucine and phenylalanine which have very important functions in the human body. Hazelnut is also a source of some important minerals such as Fe, Ca, K, K, Mg, Mn, Zn and Cu, which are necessary for physical and mental development of humans.^2^ Moreover, hazelnut varieties are rich in phytosterols known to reduce serum cholesterol and low-density lipoprotein cholesterol levels of mildly hypercholesteremic test subjects.^3^

Nut butter or spreads are spreadable products prepared by grinding nuts into a paste.^4^ Hazelnut-based butter or spreads are a mixing of hazelnut puree (*HP*), starch-based refined sugar (*SBRS*) or beet sugar, emulsifiers, stabilizers, flavor compounds (vanilla) and fillers (whey or milk powder, etc.).^5^ *SBRS* is preferred over beet sugar in many foods because of its advantages such as lightening the color of foods, accelerating caramelization and fermentation, preserving minerals and vitamin C, being softer, freezing later, positively affecting taste and being cheaper.^6^ However, studies have shown the adverse health effects of refined sugar consumption such as increasing the risk of cancer, obesity, type 2 diabetes and cardiovascular disease, damaging β cells, causing insulin resistance, dyslipidemia and lipogenesis, and increasing blood pressure.^7–10^ As consumer demand for healthier alternatives increases, interest in replacing refined sugars with healthier sweeteners has also increased. For this purpose, some artificial sweeteners (aspartame, sucralose, etc.) are used to maintain sweetness in the production of low-calorie or sugar-free products, but their safety concerns are driving demand for natural sweeteners.^11^ In this context, grape molasses (*GM*) is a healthy alternative for use as a natural sweetener in the food industry.

*GM* is a useful and important source of energy as it contains certain minerals (K, P, Ca, Mg, Fe, Mn, Cu, Zn), vitamins (Vit B1, Vit B2 and niacin), phenolic compounds and organic acids in addition to its high sugar content.^12^ Since the majority of carbohydrates are in the form of monosaccharides, it can quickly pass into the bloodstream. This makes it an important source of nutrients for infants, children, athletes and in situations where immediate energy is needed.^13^ *GM* is most commonly consumed at breakfast and is also used as a natural sweetener as a natural sweetener instead of *SBRS* or beet sugar in products such as tahini and halva.^12,14^ Furthermore, the glycemic index (GI) values of honey, syrup, and molasses vary between 1 and 60, depending on the sugar composition, and are lower than that of sucrose (GI: 65), maltose (GI: 105), maltodextrin (GI: 110), glucose, and dextrose (GI: 100). The use of molasses as a sweetener in foods will not only reduce the GI but also help to stabilize blood sugar.^15^

One of the main problems in butters from seeds and nuts is the tendency of the oil to separate and agglomerate on the surface during storage, as the liquid oil has a lower density than the ground solids. To overcome this problem, fully hydrogenated oils or other semisolid fats such as palm oil/palm oil fractions are used as stabilizers. In the absence of these stabilizers, oil separation can occur in a very short time, in some cases within a few hours to a few days.^4,16^ On the other hand, this layer of oil on the surface reduces consumer acceptance as it causes the product to solidify and makes consumption difficult.^17^ Moreover, rancidity and an unpleasant odor occur in this separated oil through lipid peroxidation.^18^ Therefore, these undesirable structural and sensory deteriorations should be prevented. To reduce oil separation and improve the texture of the product, strategies of grinding nut butter particles to a very fine size (usually <50 μm) and adding natural stabilizers are proposed.^19^ In the literature, there are many studies on the effect of factors such as packaging, storage, use of emulsifiers and stabilizers on the oxidative stability, shelf life and sensory, rheological and functional properties of oilseed or nut paste and butters,^1,4,11,16–18,20–28^ but there are limited studies on the enrichment of hazelnut products with different foods and the effect of temperature and time on oil separation in hazelnut butters.^29–31^

To our knowledge, no data are available on the evaluation of the effects of *GM* on hazelnut butter. In the present study, different amounts of stabilizer (*Stb*) and skimmed milk powder (*SMP*) were used to prevent oil separation in hazelnut butter, as well as *GM* compatible with hazelnut odor and taste instead of *SBRS* or beet sugar as sweetener. Therefore, this study aimed to determine the emulsion stability, physicochemical, nutritional and sensory properties of *GM*-fortified hazelnut butter (*GMHB*) formulations with and without *Stb* and *SMP*.

## 2 Materials and Methods

### 2.1 Materials

*HP* (Fiskobirlik Integrated Hazelnut Processing Facility-Giresun//Türkiye), *GM* (Torku Fruit Juice, Vinegar, and Molasses Production Facility- Konya/Türkiye), commercial *Stb* (Palm oil origin, AAK Akoblend-İstanbul/Türkiye) and *SMP* (Altaş Ordu Oil Industry Incorporated Company-Ordu/Türkiye) were provided by producer companies. All chemicals used in the analyses were of analytical grade.

### 2.2 Methods

#### 2.2.1 Preparation of Hazelnut Butter Formulations

One of the study’s goals was to obtain a more natural paste compared to commercial products. This subjection, in GMBH formulations, utilized natural additives such as HP, GM, SMP and a stabilizer made from palm oil that was necessary to ensure stability. The amounts of *HP*, *GM*, *Stb* and *SMP* used in *GMHB* formulations were determined to be eight formulations as a result of preliminary sensory experiments.

The primary focus of the study was on the composition of the commercial product, with the formulation designed to replace sugar content by incorporating molasses. To accurately determine the necessary additive amounts, a preliminary sensory test was conducted. The optimal blend was established based on the control sample’s color, consistency, sweetness, and a balance of roasted hazelnut and molasses flavors. After achieving the ideal ratio of *GM* and *HP*, the legally required additives were incorporated.

Each formulation was homogenized in 1000 mL glass jars for a homogeneous structure for 5 min (IKA Ultra Turrax-T18, probe S18N-19G) at 15.6 × 1000 rpm. Formulations stored at +4 °C (refrigeration chamber) until the day of analysis. Formulations for *HP*, *GM*, *Stb* and *SMP* are presented in Table 1.

**Table 1:** Design of *GMHB* Formulation Preparation

	components (%)			
*GMHB* formulations	*HP*	*GM*	*Stb*	*SMP*
A0	85.0	15.0	0.0	0.0
A1	82.5	14.5	3.0	0.0
A2	83.5	15.0	1.5	0.0
A3	82.5	14.5	1.5	1.5
B0	75.0	25.0	0.0	0.0
B1	73.0	24.0	3.0	0.0
B2	74.0	24.5	1.5	0.0
B3	73.0	24.0	1.5	1.5

#### 2.2.2 Dry Matter (*DM*) and Total Soluble Solids (*TSS*)

*DM*% was determined as a humidity analyzer having a halogen lamp heating system (Radwag, MAC 50, Poland). The *TSS* of *GM* samples was determined by a digital refractometer (Hanna HI 96800, Romania).

#### 2.2.3 pH and Titratable Acidity (*TA*)

The pH measurements of *GM* and *HP* samples were determined by a benchtop pH meter (Mettler Toledo Seven Compact S210, Switzerland). *TA* was determined by titration with 0.1 N NaOH to pH 8.1, and calculated as a percentage based on tartaric acid for *GM* and oleic acid for *HP*.^32^

#### 2.2.4 Color Measurements

Hunter L* (L* = 0, darkness; L* = 100, lightness), a* (+a*, redness; −a*, greenness), and b* (+b*, yellowness; −b*, blueness) color values were determined on the surface of samples with a color measuring device (Konica Minolta CR-410) after being calibrated as L* = 97.79, a* = −0.44, and b* = +2.04 using a calibration plate.

#### 2.2.5 Particle-Size Distribution

Particle sizes belonging to deoiled hazelnut meals were determined using the No 40 (425 μm), No 60 (250 μm), and No 140 (106 μm) of U.S.A. standard testing sieves (W. S. Tyler-Made in the USA).

#### 2.2.6 Viscosity

The viscosity of the homogeneously mixed samples was measured in cP with a viscometer (And SV-10 Sine Wave Vibro Digital Viscometer) in standard 35 mL sample cuvettes at 22.4 °C. The viscometer was used after calibrating with distilled water to 1.00 cP at 20 °C.

#### 2.2.7 Total Oil Analysis (*TO*)

The cold extraction method was used to determine the *TO* of *HP* and *GMHB*. Twenty grams homogenized sample weighed into falcon tubes was completed to 50 mL with *n*-hexane, and mixed in a shaker for 10 min at 25 °C, then centrifuged at 6000 rpm for 5 min. The procedure was repeated three times for each sample. The solvent from the *n*-hexane-oil mixture was evaporated by a rotary evaporator (Heidolph Laborota 4000, Germany, 60–120 rpm) with a vacuum pump (KNF-N810.3FT.18, 40 °C, 6–8 mbar). The amount of remaining oil was calculated as % using the weight difference.

#### 2.2.8 Free Fatty Acidity (*FFA*%)

Briefly, 2 g of the cold extracted oil samples were weighed into flasks and 10 mL of the diethyl ether-ethanol mixture (1:1 v/v) was added. Then, 2–3 drops of 1% phenolphthalein were added and titrated with 0.1 N ethanolic KOH solution until a light pink color was observed.^33^

#### 2.2.9 Peroxide Value (*PV*)

The *PV* (meq O_2_/kg oil) of the samples was determined titrimetrically according to the method described by AOAC- 965.33.^34^ Two grams of sample was mixed with 10 mL chloroform–methanol (7:3 v/v), 15 mL acetic acid and 1 mL saturated potassium iodide solutions, and the mixture was kept in the dark for 5 min. Subsequently, 75 mL distilled water and 1 mL starch solution were added to the mixture and titration was carried out with 0.02 N Na_2_S_2_O_3_ solution.

#### 2.2.10 Total Tocopherol Content (*TT*)

For *TT* analysis, 2.75 mL of toluene, 1.75 mL of 2,2′-bipyridine, and 0.25 mL of FeCl_3_·6H_2_O, respectively were added to 0.1 g of the oil obtained by using the cold extraction method, and the total volume was made up to 5 mL by adding a 95% ethanol. The absorbance value was read by a spectrophotometer at a wavelength of 520 nm after waiting for 2 min in a dark environment. The formula specified in the method was used to calculate the results.^35^

#### 2.2.11 Total Phenolic Content (*TPC*)

The extraction of phenolics was carried out following the method by Çakır et al.^36^ The colorimetric Folin-Ciocalteu method was used to determine *TPC* and the results were calculated as mg gallic acid equivalent (*GAE*)/100 g sample using the calibration curve obtained from gallic acid solutions.^37^

#### 2.2.12 Antioxidant Activity

For *DPPH* free radical scavenging activity (*DPPH*-*RSA*), an aliquot (100 μL) of the phenolic extracts of the samples mixed with 2.9 mL of *DPPH* radical solution and then incubated for exactly 30 min. Following incubation, absorbance measurements were performed at 517 nm with a spectrophotometer. The following formula was used to calculate *DPPH*-*RSA*.^32^

$$DPPH‐RSA\;\left(inhibition\%\right)=\left[1-\left({ABS}_{\mathrm{sample}}-{ABS}_{control}\right)\times 100\right]$$

#### 2.2.13 5-Hydroxymethylfurfural (*HMF*)

Briefly, samples (1 g) diluted with distilled water were precipitated with Carrez-I and Carrez-II solutions and clarified with Whatman-1 paper. Then, 1 mL of the filtrate was mixed with 2.5 mL of *p*-toluidine and 1 mL of barbituric acid and the absorbance of the red color formed in 2 min was read at 550 nm spectrophotometer (Shimadzu UV mini-1240, Japan). The formula specified in the method was used to calculate the HMF (mg/kg).^32^

#### 2.2.14 Mineral Profile

The mineral profiles of *GMHB* formulations were determined according to the NMKL 186 method.^38^ Samples were incinerated in three stages (Rise: 200 °C-20 min, Combustion: 200 °C-15 min, Cooling: 15 min) using by microwave combustion unit (Milestone, Ethos Easy Microwave Digestion System, Italy), read using ICP-MS (Thermo Scientific ICAP Q) 20 min after the plasma was formed. Three parallel readings were made for all samples. In the calibration standards’ preparation, a multielement standard solution (Chem-Lab, Belgium) with a concentration of 100 mg/kg was used as a stock standard. Calibration curves were created by taking three readings at each calibration point by QTegra software. High-purity argon gas was used as the carrier gas in the ICP-MS system. ICP-MS operating conditions are summarized below.

Spectrophotometer: Mass

RF Power: 1550 W

Plasma gas flow rate: 14 (L/min)

Auxiliary gas flow rate: 0.80 (L/min)

Nebulizer gas flow rate: 1.10 (L/min)

Sampling depth: 15 mm

Acquisition mode: Spectrum

Recurrence: 3

Spray chamber temperature: 2.7 °C

Nebulizer type: PFA

Sampler cone: Nickel cone

Analytical masses: ^9^Be,^11^B, ^14^Si, ^23^Na, ^24^ Mg, ^27^Al, ^39^K, ^44^Ca, ^51^V, ^52^Cr, ^55^ Mn, ^57^Fe, ^59^Co, ^60^Ni, ^63^Cu, ^66^Zn, ^75^As, ^77^Se, ^88^Sr, ^95^ Mo, ^107^Ag, ^111^Cd, ^121^Sb, ^137^Ba, ^205^Tl, ^208^Pb.

#### 2.2.15 Accelerated Oil Separation (*AOS*)

Analysis of *AOS* after centrifugation of *GMHB* formulations (10 g) at 2000 rpm (465*g*) at 20 °C for 10 min in a centrifuge device (Nuve NF800R), the amount of separated oil was found by proportioning the total sample weight (% w/w).^39^

#### 2.2.16 Cumulative Oil Separation (*COS*)

Equal amounts (30 g) of eight *GMHB* samples were weighed into 50 mL glass jars in three replicates (*n* = 3) and stored at 25 and 45 °C after being closed with metal lids with a lacquer layer. During 37 days of storage, *COS* was detected in 10 periods on different days (1, 4, 7, 11, 14, 18, 22, 27, 32, and 37 days) and the same jar was stored under the same conditions until the following analysis day. For *COS* analysis, the oil accumulated on the surface of the glass jars containing the *GMHB* formulations on the specified days was turned upside down on a filter paper (Whatman #4) of known tare and weighed after waiting for 10 min to absorb the oil. Then, the amount of oil separated after deducting the tare of the filter paper was determined.^40^

#### 2.2.17 Sensory Evaluation

*GMHB* formulations were prepared by homogenizing 1 L glass jars using different ratios of *HP* (73–85%), *GM* (14.5–25%), *Stb* (0–3%) and *SMP* (0–1.5%). To balance the taste, odor and structure of GMHBs were aged for 24 h before being evaluated sensorially. During sensory evaluation, a series of 8 coded samples and a control, each exactly 10 g, were presented to each panelist in random order to minimize bias. Hazelnut paste with a known formulation and the standard formulation used in the market (FİSKOBİRLİK A.Ş.-Giresun, Turkey) served as the control sample. Sensory evaluation was conducted using coded standard plastic containers with lids to prevent contamination and maintain sample integrity. Evaluators were informed about the product’s compliance with current specifications, its advantages, and its similarities and differences compared to the current product. Panelists were then asked to participate in a prepreference test (Preference Tests) to determine their liking of the *GMHB* samples. After than panelists approved the *GMHB* samples, the analysis was initiated. Panelists were also asked to express their preferences as part of the *GMHB* development process. Evaluators were informed that no harmful additives were used in the product, and their consent was obtained before participating. *GMHBs* were evaluated by eight panelists (6 male and 2 female) trained in food engineering using a 1–5 point hedonic scale (1–5 points, 5: like extremely, 4: like slightly, 3: neither like nor dislike, 2: dislike slightly, 1: dislike extremely) for color, taste, odor, spreadability, homogeneous structure, mouthfeel and overall acceptability. Before starting another presentation, the aftertaste in the mouth of *GMHBs* was removed with bread slices.

According to the aforementioned criteria, *GMHB* formulations received low scores if the color was dull and lighter or much darker than the control. Bright and similar to the control sample earned higher scores. In terms of closeness and superiority to the control sample, *GMHBs* with dominant hazelnut aroma and molasses taste harmony, spreadability, and a smooth, homogeneous structure received high scores. To ensure a consistent environment, evaluations were carried out in a sensory testing laboratory setting under controlled lighting and temperature conditions.

#### 2.2.18 Statistical Analysis

The *GMHB* formulations were prepared in duplicate, and the physicochemical experimental procedures for *GMHB* were conducted in triplicate. Sample analyses for *GM* and *HP* were performed in duplicate, with the results expressed as mean values ± standard deviation (*SD*). Only the experimental data regarding the compositional properties of *GMHB* were analyzed using one-way *ANOVA* with *MINITAB*-*18* software. Tukey’s multiple comparison test was utilized to identify statistically significant differences between means (*P* < 0.05).

To assess the stability and shelf life of *GMHB* samples based on the average *COS* value from three replicates, a stability study plan was developed using software. This plan utilized regression analysis, focusing on Time (*T*) and Sample (*S*), and was analyzed using *MINITAB*-*18* software.

## 3 Results and Discussion

### 3.1 Characteristics of *GM* and *HP*

Table 2 shows some characteristics of *GM* and *HP*. *GM* had 72% Total soluble solids (*TSS*) and dry matter (*DM*) content was lower than *HP*. Titration acidity (*TA*) for *GM* and *HP* were 0.403% (as tartaric acid) and 0.863% (as oleic acid), respectively, and the pH of *HP* (6.11) was higher than that of *GM* (5.28). Total oil, *FFA*, *PV*, and *TT* values of *HP* varied between 63.50–65.00%, 1.035–1.072%, 9.85–9.94% meq O_2_/kg oil and 350–395 mg/kg, respectively. *GM* (746 cP) was more viscous than *HP* (510 cP), while *HMF* content was lower in *GM*. Although *TPC* was lower in *GM* compared to *HP*, *GM* showed higher *DPPH-RSA*. The viscosity values for *GM* and *HP* were 746 cP and 510 cP respectively, while the values for Hunter L*, a*, and b* were higher for *GM*. On the other hand, the distribution and sizes of *HP* particles were classified as 425–280 μm (63.03%), 250–106 μm (35.09%), and <106 μm (1.88%) using the USA standard testing sieves. These findings for *HP* and *GM* were consistent with those reported in previous studies.^36,41,42^

**Table 2:** Some Physicochemical Properties of *GM*, *HP*, and *GMHB* Formulations^a^

			*GMHB* formulations^b^							
physicochemical properties	*GM*	*HP*	A0	A1	A2	A3	B0	B1	B2	B3
DM (%)	75.05 ± 0.64	98.91 ± 0.08	95.08 ± 0.34^BC^	98.66 ± 0.35^A^	95.87 ± 0.88^B^	94.99 ± 0.54^B‑D^	93.43 ± 0.53^CD^	93.51 ± 0.59^CD^	92.91 ± 0.33^D^	93.37 ± 0.26^CD^
TSS (%)	72.00 ± 1.13	-	-	-	-	-	-	-	-	-
pH	5.28 ± 0.02	6.11 ± 0.02	5.77 ± 0.01^C^	5.82 ± 0.01^BC^	5.86 ± 0.02^B^	5.91 ± 0.01^A^	5.82 ± 0.00^BC^	5.85 ± 0.01^B^	5.86 ± 0.01^B^	5.91 ± 0.00^A^
TA (%)	0.403 ± 0.01^†^	0.863 ± 0.04^‡^	1.295 ± 0.05^A^	1.155 ± 0.05^AB^	1.015 ± 0.05^BC^	0.945 ± 0.05^C^	1.295 ± 0.05^A^	1.190 ± 0.00^AB^	1.085 ± 0.05^BC^	1.015 ± 0.05^BC^
total oil (%)	-	64.25 ± 1.06	-	-	-	-	-	-	-	-
FFA (%)	-	1.053 ± 0.03	1.007 ± 0.01^D^	1.014 ± 0.00^CD^	1.152 ± 0.01^A^	1.124 ± 0.00^A^	0.982 ± 0.02^D^	1.025 ± 0.02^B‑D^	1.066 ± 0.02^B^	1.060 ± 0.01^BC^
PV (meq O_2_/kg oil)	-	9.896 ± 0.06	9.324 ± 0.66	9.925 ± 0.04	10.986 ± 0.00	9.777 ± 0.14	10.740 ± 1.09	10.377 ± 0.79	10.400 ± 0.67	10.294 ± 0.60
HMF (mg/kg)	18.76 ± 2.2	24.38 ± 1.9	14.13 ± 0.29^BC^	14.37 ± 1.45^BC^	18.00 ± 0.28^A^	15.51 ± 0.28^B^	12.81 ± 0.63^CD^	11.88 ± 1.16^D^	12.84 ± 0.62^CD^	12.82 ± 0.60^CD^
TT (mg/kg)	-	370.20 ± 28.4	326.53 ± 1.6^AB^	337.56 ± 25.3^A^	317.10 ± 8.2^AB^	303.57 ± 1.0^AB^	323.52 ± 13.5^AB^	285.98 ± 1.4^B^	306.70 ± 3.6^AB^	317.69 ± 2.5^AB^
TPC (mg GAE/100 g)	297.57 ± 14.9	840.92 ± 0.7	525.54 ± 36.3^AB^	629.77 ± 6.6^A^	520.36 ± 37.2^AB^	493.46 ± 23.3^B^	505.80 ± 2.8^B^	552.04 ± 28.1^AB^	511.23 ± 27.2^B^	517.86 ± 24.6^AB^
DPPH-RSA (%)	54.74 ± 2.2	34.66 ± 0.5	48.03 ± 0.2	48.01 ± 0.3	45.21 ± 4.2	47.60 ± 1.3	49.89 ± 2.6	48.44 ± 0.6	47.57 ± 0.31	47.34 ± 0.70
hunter color values										
hunter L*	23.10 ± 0.45	33.59 ± 0.01	24.67 ± 0.39^D^	24.62 ± 0.39^D^	24.33 ± 0.38^E^	23.93 ± 0.38^F^	25.72 ± 0.34^A^	24.92 ± 0.33^C^	25.32 ± 0.34^B^	24.92 ± 0.33^C^
hunter a*	2.13 ± 0.71	5.92 ± 0.014	2.70 ± 0.60^B^	2.68 ± 0.61^B^	2.66 ± 0.59^B^	2.61 ± 0.59^B^	3.07 ± 0.53^A^	2.97 ± 0.52^A^	3.02 ± 0.53^A^	2.97 ± 0.52^A^
hunter b*	–0.19 ± 0.10	8.94 ± 0.002	1.18 ± 0.09^D^	1.13 ± 0.08^E^	1.18 ± 0.08^D^	1.14 ± 0.08^E^	2.09 ± 0.08^A^	2.01 ± 0.07^C^	2.05 ± 0.07^B^	2.01 ± 0.07^C^
particle size (μm)	-	260.3 ± 159.7	-	-	-	-	-	-	-	-
viscosity (cP)	746 ± 7.2	510 ± 20	599 ± 35^D^	1332 ± 295^D^	839 ± 97^D^	745 ± 60^D^	4232 ± 683^B^	10,124 ± 1689^A^	3002 ± 381^BC^	2027 ± 101^CD^

a The average values given as Mean ± SD (Standard deviation) were obtained from two replicate samples for HP and GM and three replicate samples for GMHB. DM: Dry matter; TSS: Total soluble solids; TA: Titratable acidity; FFA: Free fatty acidity; PV: Peroxide value; TT: Total tocopherol; TPC: Total phenolic content. Calculated as †: tartaric acid, ‡: oleic acid.

b Means shown with the same letter are not statistically different from each other (*P* < 0.05).

### 3.2 Physicochemical and Nutritional Properties of *GMHB* Formulations

Some physicochemical and bioactive properties of *GMHB* formulations prepared using different ratios of *GM*, *HP*, *Stb*, and *SMP* are presented in Table 2. As a result of decreasing *HP* and increasing *GM* and *Stb* ratios in *GMHB* formulations, Group-A samples (94.99–98.66%) had higher *DM* amounts compared to Group-B samples (92.91–93.51%). In all *GMHB* formulations, pH decreased with the addition of *GM* and *Stb* and increased slightly with the inclusion of *SMP*. The highest pH values were determined in samples A3 and B3. Consistent with the pH values, the highest *TA* (as % oleic acid) was determined in the A0 and B0 samples and the lowest values in the samples with *SMP*. These results showed that the use of *SMP* in *GMHB* formulations significantly affected acidity. *FFA* and *PV* are widely used quality indicators for oils and fats and refer to the degree of hydrolysis and oxidation, respectively.^29^ In the present study, the addition of *Stb* and *SMP* increased the *FFA* values of *GMHB* samples and the highest values were found in samples A2 and A3 (*P* < 0.05). Although Group-B samples had generally higher *PV* values than those in Group-A, there was no significant difference between the *PV* values in the range 0.934–10.986 mequiv O_2_/kg for all *GMHB* samples (*P* > 0.05). Nevertheless, the insignificant differences in the *PV* values of Group-B and Group-A can be attributed to the different ratios of water-containing *GM* in the formulations. Similarly, Abegaz et al.^43^ reported that the *PV* of peanut butter increased with the addition of water (2–5 g H_2_O/100 g) and decreased with the addition of sugar (4 g sucrose/100 g). Adhikaray^44^ also observed that the effect of temperature on the oxidation and rancid flavor in kinds of peanut butter was more effective than that of moisture.

Regarding *HMF* content, the *GMHB* mixtures in Group-A had higher *HMF* content compared to Group-B, with the highest content in sample A2 (*P* < 0.05). Group-B *GMHB* formulations were found to have similar (*P* > 0.05) *HMF* content. On the other hand, *HMF* contents were not reflected in the formulations in proportion to *GM* and HP mixing ratios. The expected *HMF* content was found to deviate by approximately 27–48% in all formulations, with this loss being higher in the *GMHB* samples of Group-A with a high *HP* ratio than in Group-B. These differences can be explained by the fact that Carrez I and Carrez II solutions used for clarification during analysis form complexes with proteins involved in *HMF* formation.^45^

Total tocopherol (*TT*) content of *GM*-enriched hazelnut butters generally varied within similar (*P* > 0.05) limits (285.98–337.56 mg/kg), with the highest and lowest *TT* content found in samples A1 and B1, respectively (*P* < 0.05). In a previous study, hazelnut paste enriched with green coffee extract was reported to have similar *TT* content during 3 months of storage at 25 °C.^30^

In *GMHB* formulations, decreasing *HP* and increasing *GM* reduced the amount of *TPC*. Group A samples generally yielded higher *TPC* than Group B samples. However, the highest *TPC* for both groups was found in formulations with 3% *Stb* addition (*P* < 0.05).

The main point is that although the amount of *TT* and *TPC* showed a statistically insignificant decrease based on the mixing ratio, a slight increase was observed in some mixtures. This may be because of the *TT* and *TPC* contributions present in palm oil. A study on the topic reported that palm oil is rich in *TT* (1026–6700 mg/kg) and *TPC* (66–176 mg GAE/kg).^46^

The inhibition rate of *DPPH* (2,2-diphenyl-1-picrylhydrazyl) radical for all *GMHB* samples was in the range of 45.21–49.89%. Although the presence of *Stb* and *SMP* reduced *DPPH* activity, no significant difference was found among the formulations (*P* > 0.05). However, it was slightly higher in Group B, where the amount of molasses was higher, compared to Group A samples. Consistent with our data, Kaya et al.^47^ found that the addition of sesame and peanut butter to grape and carob molasses increased the *TPC* and *DM* of the mixtures, while decreasing the water activity (*a*_w_), thus maintaining their biological value for a long time without preservatives. Alqahtani et al.^11^ reported that *TPC* and antioxidant activity of peanut butters enriched with date syrup (dibs) as a natural sweetener increased linearly with increasing dibs concentration. Şimşek et al.^30^ reported that fortification of hazelnut paste with green coffee extract increased TFM and antioxidant activity while decreasing *FFA* and *PV*.

*HP* had higher Hunter L*, +a*, and +b* values compared to *GM*, but this was interestingly reflected in the *GMHB* mixtures. Hunter L*, a* and +b* values of *GMHB* samples increased with decreasing *HP* and increasing *GM* content, but generally decreased with the addition of *Stb* and *SMP*. The highest Hunter L* and +b* values were determined in B0 sample with %75 *HP* and %25 *GM*. Hunter + a* values in Group-B formulations were similar (*P* > 0.05) but higher (*P* < 0.05) than those in Group-A samples. Similarly, Hathorn and Sanders^48^ observed that +a* (redness) and darkness (−L* value) increased as the skin ratio used in peanut pastes increased. In general, the pellicle is added to the purees to thicken their color, increasing the aflatoxin load in the pastes. Therefore, using *GM* to adjust the color of pastes may reduce the risk.

The viscosity of the *GMHB* formulations increased with increasing *GM* and *Stb* content, this increase was more pronounced in Group-B samples than in Group-A (*P* < 0.05). This can be attributed to the higher *GM* content in Group-B samples (24–25%) compared to those in Group-A (14.5–15%). In addition, the addition of *Stb* alone increased the viscosity of the formulations, while the use of *Stb* and *SMP* together led to a decrease in viscosity. In this regard, the highest viscosity values in both groups were in samples A1 (1332 cP) and B1 (10124 cP) with 3% *Stb* (*P* < 0.05). On the other hand, the viscosity of formulations A3 and B3, in which *Stb* and *SMP* were used together and in equal ratios, was relatively lower than that of the samples containing only 1.5% *Stb* (A2 and B2). These results suggest that *SMP* can be an effective solution for adjusting the viscosity of nut butters. Similarly, Yeh et al.^21^ reported that *Stb* was required alongside milk powder samples to prevent oil separation, but excessive *Stb* resulted in harder peanut butters. Totlani and Chinnan^23^ declared that *Stb* ratios (0–2%) and storage conditions (3 months at 35 and 26 °C) as important factors affecting the consistency of peanut butter. In the present study, third group *GMHB* formulations (coded C) were also produced using 65% *HP*–35% *GM*, but these formulations were excluded from the experimental plan due to the fact that the high *GM* ratio increased viscosity and oil separation as well as forming a plastic structure during homogenization. Maskan and Göğüş^20^ associated the firmness (*a*_w_ < 0.15) or softness (*a*_w_ > 0.85) of pistachio paste with low or high *a*_w_. While this may be partially true, the increased hardness and gel-like consistency observed in Group B-*GMHB* formulations with high water content may be attributed to protein gelation, which may result from the protein-sugar interaction as well as changes in protein solubility and isoelectric point due to pH reduction.

### 3.3 Macro and Microelement Contents of *GMHB*

#### 3.3.1 Macroelements

Table 3 shows that among some of the macro elements determined in 8 different GMHBs, K was the highest (in the range of 12,755–14,350 mg/kg), followed by Ca (2465–3005 mg/kg), Mg (1899–2376 mg/kg), Na (884–1664 mg/kg) and Si (516–773 mg/kg). Among all formulations, B3 had the highest Na and Ca contents and A2 had the highest Mg and Si contents (*P* < 0.05). On the other hand, the K, Na and Ca contents of Group-B formulations with high *GM* content were higher compared to Group-A (Table 3). The K, Na, Ca and Mg content of *GMHB* samples decreased slightly with the addition of 3% *Stb*, while a slight increase was observed with the fortification of *SMP*. However, the presence of *Stb* and *SMP* had no significant effect on the Si content (*P* > 0.05).

**Table 3:** Contents of Macro and Micro Elements of *GMHB* Formulations^a^,^c^

		*GMHB* formulations^b^							
	minerals (mg/kg)	A0	A1	A2	A3	B0	B1	B2	B3
macroelements	K	13,479.1^AB^	13.296.0^AB^	14.198.4^A^	13.655^AB^	13.640.9^AB^	12.755.3^B^	13.907.0^AB^	14.350.1^A^
	Ca	2707.2^B–D^	2617.4^CD^	2780.7^A–C^	2916.2^AB^	2637.5^CD^	2464.8^D^	2638.0^CD^	3005.4^A^
	Mg	2313.9^AB^	2269.6^AB^	2376.1^A^	2272.9^AB^	2010.1^C^	1899.1^C^	2096.1^BC^	2098.5^BC^
	Na	901.2^C^	883.5^C^	1013.7^C^	1046.8^C^	1589.4^AB^	1444.1^B^	1496.5^AB^	1663.7^A^
	Si	689.7^AB^	690.7^AB^	772.7^A^	626.4^A–C^	515.5^C^	554.0^BC^	613.4^A–C^	590.6^BC^
microelements	Fe*	113.29	109.17	115.87	110.43	106.14	98.09	105.19	106.48
	Mn	82.53^A^	79.78^AB^	83.48^A^	78.84^A–C^	70.02^CD^	65.71^D^	72.71^B–D^	70.84^B–D^
	B	62.20^AB^	61.07^A–C^	62.67^A^	59.18^A–C^	54.71^CD^	51.63^D^	56.10^A–D^	55.49^B–D^
	Zn	51.01^A^	47.81^A–C^	49.82^AB^	47.44^A–C^	41.74^CD^	37.64^D^	43.52^B–D^	41.78^CD^
	Cu	37.89^AB^	38.01^AB^	39.46^A^	36.94^A–C^	33.045^CD^	31.49^D^	34.31^B–D^	33.51^CD^
	Al*	11.80	11.70	13.47	11.25	12.17	11.77	11.39	10.90
	Sr	8.019^AB^	7.887^AB^	8.301^A^	7.943^AB^	7.338 ^BC^	6.871^C^	7.466 ^A‑C^	7.642 ^A‑C^
	Ni	7.698^A^	7.588^A^	7.709^A^	7.364^AB^	6.687 ^AB^	6.449 ^B^	6.834 ^AB^	7.142 ^AB^
	Ba	6.031^AB^	6.210^A^	6.293^A^	5.978^A–C^	5.323 ^CD^	4.910 ^D^	5.326 ^CD^	5.367 ^B‑D^
	Cr	1.414^A–C^	1.474^AB^	1.597^A^	1.419^A–C^	1.242 ^C^	1.270 ^BC^	1.344 ^BC^	1.337 ^BC^
	Ti*	0.881	0.848	0.902	0.966	0.826	1.448	0.862	0.918
	Co	0.743^A^	0.685^AB^	0.713^AB^	0.688^AB^	0.624 ^BC^	0.5614^C^	0.638 ^BC^	0.622 ^BC^
	Mo*	0.221	0.219	0.239	0.232	0.228	0.185	0.219	0.227
	Se*	nd	nd	0.0063	nd	nd	0.0019	0.0024	0.0078
	Pb	0.060^A^	0.054^AB^	0.053^AB^	0.054^AB^	0.044^A–C^	0.033^C^	0.038^BC^	0.033^C^

a The average values given as Mean were obtained from two replicate samples for GMHB.

b Means shown with the same letter are not statistically different from each other (*P* < 0.05).

c nd: not detectable.

#### 3.3.2 Microelements

The contents (mg/kg) of 14 microelements determined in *GMHB* formulations are presented in Table 3. Fe was the most abundant microelement in *GMHB* samples with a content ranging from 98.09–115.87 mg/kg. The decreasing hierarchy for the microelement profile of *GMHB* samples is Fe > Mn > B > Zn > Cu > Al > Sr > Ni > Ba > Cr > Ti > Co > Mo > Se. The contents of Fe, Al, Ti, Mo and Se were similar in all *GMHB* formulations. However, increasing *GM* ratio in *GMHB* formulations generally decreased the amounts of Mn, B, Zn, Cu, B, Sr, Ni, Ba, Cr and Co. In this context, Group-A samples with higher *HP* ratio are richer in microelements compared to Group-B. On the other hand, six of the 26 minerals read by *ICP-MS* were not detectable. Three of these minerals are Be, Sb and V presented in trace elements and the others are Tl, As and Cd, which are known to be toxic to living organisms. The fact that toxic elements were not detected in the *GMHB* can be considered positive. Of the toxic minerals mentioned above, only Pb was found between 0.033–0.060 mg/kg in *GMHB* samples. In addition, high *GM* content in Group-B samples decreased the amount of Pb compared to Group-A. Şimşek et al.^12^ reported that total ash in 25 grape molasses was between 3.57–3.83%, and some minerals such as K, Ca, Mg, P, Na, Fe, Zn, Cu and Mn were determined between 885–978, 124–139, 67–81, 74–87, 30–37, 13.6–15.8, 0.11–0.14, 0.35–0.44, 0.56–0.72 in mg per 100 g, respectively. In addition, previous studies reported that hazelnut has a total ash content between 1.87–2.82% and contains minerals such as Na (2–3.8 mg), Ca (65–328 mg), Mg (2144–224 mg), P (202–370 mg), K (382–1470 mg), Mn (2.4–10 mg), B (13–23.87 mg), Co (0.47–0.82 mg), Cr (0.22–0.52 mg), Cu (17–32.23 mg), Fe (31–51.60 mg), Li (0.035–0.042 mg), Ni (1.15–2.27 mg), Se (0.96–1.39 mg) and, Zn (22–44.03 mg) in varying amounts depending on the hazelnut variety.^4,49^ Considering the literature data given above, the mineral contents of *GMHB* formulations are consistent with those of *GM* and *HP*, although there are some differences (increase in K, Ca, Mg, Na). It can also be stated that *HP* is more effective than *GM* on the rich Si content in *GMHB* formulations. The amount of minerals in herbal products and foods varies according to many factors. The type and composition of hazelnut and grape, molasses and puree production methods, molasses extraction conditions, clarifiers added to molasses, and contamination during production may have played an important role in the change of mineral profile.

### 3.4 Emulsion Stability of *GMHB* Formulations

Oil separation is a significant quality issue in oilseed pastes, negatively impacting their visual appeal and altering their appearance, spreadability, and texture during long-term storage. As oil migrates to the top and solid particles settle at the bottom, two distinct layers form, resulting in a hard and uneven paste. This separation diminishes product quality, affects consumer acceptance, and ultimately reduces marketability, harming the producer’s interests.^50^

Emulsion stability of *GMHB* samples were evaluated using accelerated oil separation (*AOS*) and cumulative oil separation (*COS*) methods. The *AOS* values of *GMHB* mixtures ranged from 8.63% to 18.69%, and the highest (*P* < 0.01) *AOS* was observed in the *Stb*-free samples (A0 and B0) in both groups (Figure 1). In *GMHB* formulations, an increase in the proportion of *GM* increased *AOS* regardless of *Stb* and *SMP*.

**Figure 1:**
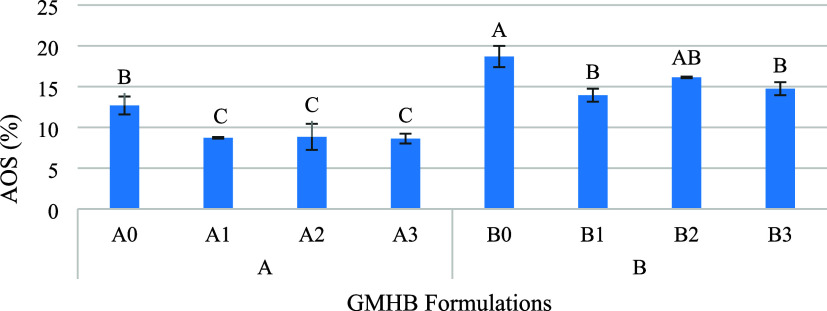
*AOS* values of *GMHB* formulations (*n* = 2)^§^. §: Means shown with the same letter are not statistically different from each other (*P* < 0.01).

The *AOS* (8.63–8.84%) of Group-A samples (A1, A2, and A3) containing *Stb* and *SMP* was similar (*P* > 0.05) and lower compared to Group-B (13.95–18.69%) (Figure 1). In fact, soluble sugars are expected to act similarly to stabilizers by adsorbing fats and oils.^19^ Ereifej et al.^40^ found that the addition of carbohydrates including sugar powder (%1) increased the emulsion stability of halva stored at 25 and 40 °C. In a previous study, Tounsi et al.^51^ found that the addition of 50% carob molasses (60 °Brix) was effective in preventing oil separation in sesame paste and attributed this to the emulsifying capacity of carob molasses, which is mainly associated with sugar molecules or Maillard reaction products. Alpaslan and Hayta^14^ attributed the dose-dependent increase in emulsion stability of sesame paste by the inclusion of grape molasses to the ability of molasses to prevent oil droplets from coalescing into larger oil droplets that tend to separate from the mixtures. However, in the nut butter system, protein, carbohydrates and other components of the colloid exist in a dynamic equilibrium ratio; when this balance is disturbed, the stability of the system is adversely affected.^19^ In this context, the increase in *AOS* with increasing *GM* in Group-B samples can be attributed to the decrease in protein content as a result of the low HP content in these samples and the disruption of the dynamic equilibrium in the product.

At the beginning of the storage period, the *COS* values for the A0 and B0 samples ranged from 1.01% to 2.09% and from 2.34% to 3.62%, respectively. After 37 days of storage at 25 °C, the *COS* values increased to 24.38% for A0 and 39.20% for B0. When the storage temperature was raised to 45 °C, these values further increased to 40.67% for A0 and 42.35% for B0. Consequently, raising the storage temperature led to increased phase separation over the same storage period.

Similarly, Gills and Resurreccion^52^ found that storage of peanut butter stabilized with palm oil at 30 and 45 °C resulted in greater oil separation compared to storage at 21 °C. In contrast to our findings, Safaei et al.^27^ found that the emulsion stability of peanut butter during 60 days of storage was significantly improved by enrichment with date paste, but the storage temperature of 45 °C had a much better effect than 25 °C. Mohd Rozalli et al.^18^ emphasized that oil separation in additive-free peanut butter was minimal (0.35%) for 16 weeks at 10 °C. They also found that oil separation was stable at 25 and 35 °C for the first 2 weeks, but increased between 2 and 10 weeks and reached 21% at the end of 16 weeks in the same study. Yao et al.^28^ reported that the addition of peanut protein-flaxseed gum conjugates (1–6%) decreased the oil separation rate of sesame paste by 1.72%–5.58% and increased the shelf life during two months of storage at room temperature. Praveen et al.^26^ reported that treatment with curcuminoid removed turmeric oleoresin (*CRTO*), *CRTO* oil and curcuminoids, which are byproducts of *Curcuma longa*, significantly reduced oil separation in peanut butters during five months storage at different conditions (27 °C and 65% RH; 37 °C and 95% RH).

Furthermore, among the *COS* ratios of *Stb*-added *GMHB* formulations stored at 25 °C, in sample A1 with 3% *Stb*, no oil separation was observed over 37 days of storage. In contrast, the *COS* of A2 (with 1.5% *Stb*) and A3 (with 1.5% *Stb* + 1.5% *SMP*) were 0.15% and 0.70%, respectively. On the other hand, the initial (day 0) *COS* values of formulations B1, B2, and B3 varied between 0.1% and 0.5% and increased by the end of the storage period (day 37) to 4.46%, 5.85%, and 11.18%, respectively. Additionally, it was found that the addition of *SMP* did not affect the *COS* in the mixtures of both groups stored at 25 and 45 °C. In agreement with our findings, studies conducted by various researchers have shown that the addition of *Stb* can prevent oil separation in nut butters. Yeh et al.^21^ reported that peanut butter with the lowest *Stb* content had the highest oil separation and that soybean flour was more effective than *SMP* in preventing oil separation. Totlani and Chinnan^23^ reported that adding 1–2% *Stb* prevented oil separation in peanut butter for up to 3 months at 35 °C. According to Zuzarte et al.,^53^ peanut butter that contains hydrogenated vegetable oils with 50% monoglyceride or triglyceride was showed maximum oil separation after 60 days of storage at 30 °C. Meanwhile, Aryana et al.^39^ discovered that adding non-hydrogenized palm oil to peanut butter can increase its oil-holding capacity, especially when stored at 0 °C, and the oil-holding capacity can increase by up to 18%.

In all *Stb*-added *GMHB* formulations, *COS* increased with longer storage times at 45 °C and was higher than that stored at 25 °C. By the end of the storage period (37th day), *COS* in A1 (0.23%) was significantly lower than in A2 (18.11%) and A3 (21.37%) samples. Conversely, the *COS* in formulations B1 (14.03%), B2 (26.86%), and B3 (33.23%), which had higher *GM* content, was greater than that in Group A over the same storage period. During *HP* production, small amounts of hazelnut oil are added externally to increase fluidity, resulting in oil separation (*AOS* or *COS*) in the final product. To overcome this problem, rested and deoiled *HP* can be used. However, if high-fat *HP* or water-containing sweeteners such as *GM* are used in production, *Stb* or water binders such as protein or starch-based products should be added to reduce *AOS* or *COS*. As a result, factors such as nut variety, oil content, natural emulsifiers (lecithin, mono- and diglyceride, proteins) and emulsion capacity of non-*Stb* additives, grinding degree (particle sizes), storage temperature and time may be effective in oil separation in *Stb*-free pastes. The results of the present study showed that the amounts of *GM*, HP and *Stb* as well as the storage temperature (*T*), time (*t*) and especially the *GM*/*HP* ratio play an important role in preventing oil separation.

**Table 4:** Regression Equations That Describe the Relationship between *T* and *COS* in *GMBH* Formulations^a^

*GMHB* samplesll	*COS* (%)
A0–25	=0.792 + 0.6312 × *T*
A1–25	=0.000 + 0.0000 × *T*
A2–25	=0.076 + 0.0027 × *T*
A3–25	=0.164 + 0.0159 × *T*
B0–25	=3.530 + 0.9884 × *T*
B1–25	=0.011 + 0.1185 × *T*
B2–25	=0.643 + 0.1428 × *T*
B3–25	=0.952 + 0.2828 × *T*
A0–45	=2.512 + 1.0528 × *T*
A1–45	=0.068 + 0.0049 × *T*
A2–45	=0.092 + 0.4840 × *T*
A3–45	=–0.107 + 0.5649 × *T*
B0–45	=5.797 + 1.0252 × *T*
B1–45	=0.672 + 0.3646 × *T*
B2–45	=2.469 + 0.6800 × *T*
B3–45	=1.697 + 0.8691 × *T*

a *T*: Times (Day).

**Figure 2:**
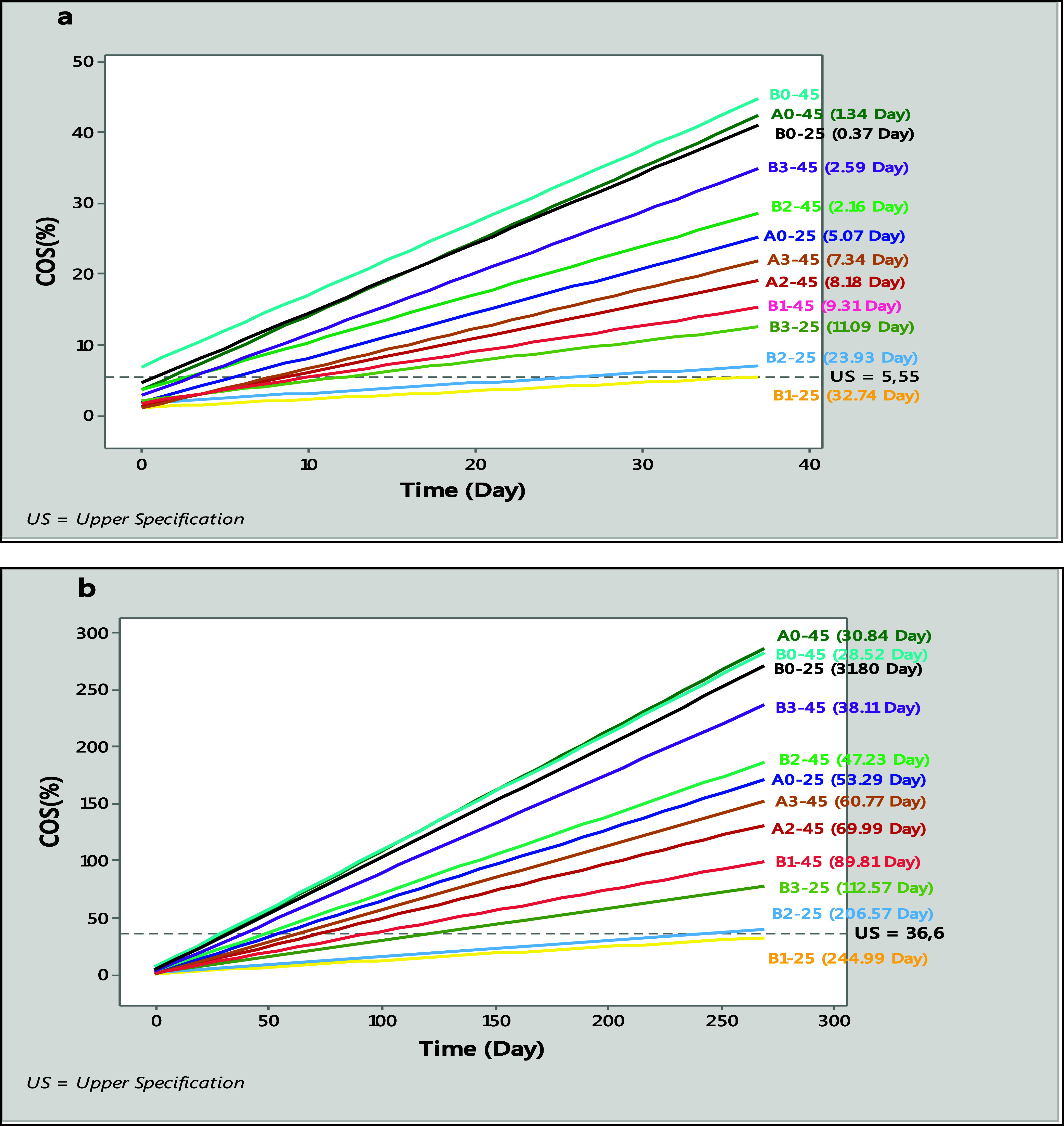
Shelf life plots of the *GMHB* formulations based on *COS*% for both the minimum (a) and maximum (b) *US* values at 25 and 45 °C (*R* ^2^: 99.68%, (a) *US* = 5.55, (b) *US* = 36.6).

In the stability study, the *ANOVA* results for the *COS* showed that the *GMHB* samples (*S*), time (*T*), and their interaction (*SxT*) were statistically significant in affecting the mean *COS* values at a *P*-value of less than 0.0001 (Appendix 1). A summary of the model, which includes the *R*-squared (*R*-*sq*) and adjusted *R*-squared (*R*-*sq*(*adj*)) values, can be found in Appendix 2. Furthermore, the specific coefficients, standard deviations, *T*-values, and *P*-values are provided in Appendix 3, while the plots for the residual values of *COS* (%) can be found in Appendix 4. The *R* ^*2*^ value of the regression equations for both Upper Specification (*US*) limits in the model study was 99.68% (Appendix 2). Also, the *P-*values for coefficient samples (*S*) ranged from 0.0001 to 0.446, with the sample and time interaction (*SxT*) term being more significant (*P* < 0.0001) (Appendix 3). Additionally, the stability study yielded the mathematical models presented below, which can reveal the shelf life according to the Upper Specification (*US*) limits for *COS* data using the *T* variable (Table 4).

**Y** = **β**_**0**_ **+ β**_***i***_ ***X***_***i***_ where *Y* = *COS*% for samples; β_0_, and β*_i_* = regression coefficients for the intercept and linear, respectively; *X_i_* (*T*) = the independent variable as storage time (day).

To develop a model for predicting shelf life based on oil stability, we used the average values of the highest and lowest oil release data from the samples collected at the end of the trial (on the 37th day) as the *US*. These limits were 5.55% and 36.6% (Figure 2a,b).

The highest observed shelf lives were for B1–25 (32.74 days), B2–25 (23.93 days), and B3–25 (11.09 days), excluding group A (A1–25, A2–25, A3–25, and A1–45), which had a *COS* of less than 1% at the end of the 37th day. Conversely, the shortest shelf lives were recorded for B0–25 (0.37 days) and A0–45 (1.34 days) (Figure 2a,b). On the other hand, the highest mean oil release value (36.6%) was achieved over longer periods, specifically 245 days for B1–25, 206.6 days for B2–25, and 112.6 days for B3–25.

The mean response slopes for batches A1–25, A2–25, A3–25, and A1–45 at both the 5.55% *US* and 36.6% *US* levels were not significantly greater than zero. Additionally, the mean response for batch B0–45 at time zero was not significantly less than 5.55% *US*. As a result, we could not predict the shelf life for eight batches.

Concerning the subject, Zuzarte et al.^53^ established 4% as an acceptable threshold for an arbitrary level of oil separation in peanut butter based on the soft to semisolid consistency of the product and the lack of obvious oil accumulation.

### 3.5 Sensory Evaluation

The sensory evaluation scores of *GMHB* samples by the panelists are shown in Figure 3. Regarding the color scores, the panelists preferred the A3 and B3 formulations of *GMHB* due to the lighter color imparted by the *SMP*.

**Figure 3:**
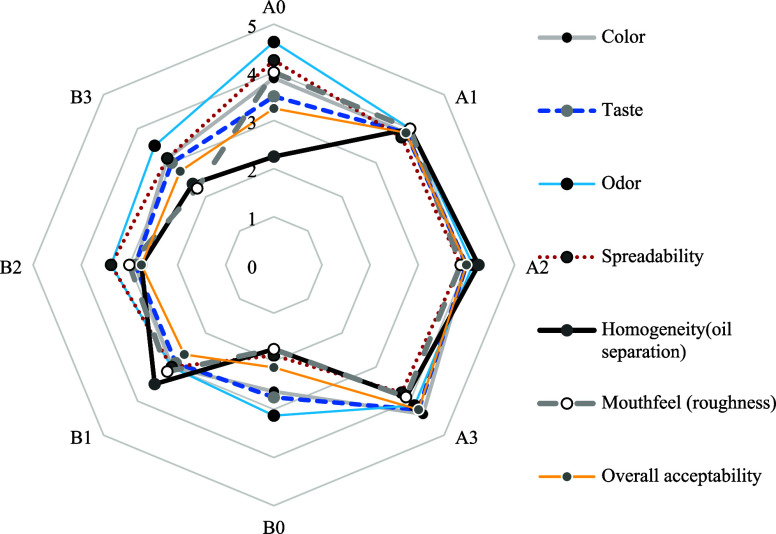
Sensory evaluation of GMHB formulations.

Similar results were reported by Khan et al.^25^ who reported that milk powder supplementation of peanut butter at different concentrations (5–15%) improved sensory color scores by presenting lighter color. On the other hand, Group-A samples with lower *GM* content had higher color scores. Among the eight formulations, A3 (4.38) and A2 (4.00) received the highest scores for taste due to their well-balanced flavor profiles. However, the higher *GM* content in Group-B formulations resulted in increased sweetness, a strong molasses taste and a slight suppression of the hazelnut flavor compared to Group-A samples. Another important factor to consider is that foreign tastes, such as woody, crusty, astringent, and bitter flavors were not detected in *GMHB* samples due to the use of *HP* with lower skin content. Likewise, Hathorn and Sanders^48^ found that peanut paste can have an undesirable taste if the pellicle ratio is more than 5%.

Panelists perceived the hazelnut odor to be more pronounced in Group-A formulations than in Group-B and this was positively reflected in the odor scores. Accordingly, Group-B samples had lower odor scores compared to Group-A, with the highest and lowest odor scores being in samples A0 and B1, respectively. The presence of *SMP* had a positive effect on the odor scores of Group-B samples, while this effect was limited in Group-A. On the other hand, A1 and B1, which had the highest *Stb* ratio, were the least accepted samples in terms of odor parameter in both groups.

Panelists evaluated the consistency of *GMHB* mixtures based on criteria such as spreadability, homogeneity (oil separation), and mouthfeel. Group-A samples had a more spreadable and noncaking consistency than those in Group-B, with the highest score in sample A0 with high HP but without *Stb* and *SMP*. B0 had the lowest level of spreadability, while B2 scored higher than B1 and B3. The correct *GM* and HP ratio can explain the difference in scores of A0 and B0 in terms of spreadability. If the ratio of HP and *GM* is not appropriate, increasing *Stb* and *SMP* in equal proportions can effectively improve the structure and spreadability of B formulations. In a similar study, the addition of 12.5% date syrup to peanut butter was reported to provide the best balance of textural properties, contributing to a product that is easy to spread, firm enough to hold its shape and not overly sticky.^11^

In the present study, the homogeneity (oil separation) scores clearly demonstrated the positive effects of *Stb* and *SMP* use in terms of improving texture, providing homogeneous structure and preventing oil separation (phase separation). The lowest homogeneity scores (visual texture assessment) were observed in A0 and B0 samples without *Stb*. Moreover, increasing the *GM* ratio decreased the homogeneity scores of *GMHB* mixtures. Regarding mouthfeel, the *GMHB* mixtures in Group-A were more appreciated by the panelists, with the highest scores for samples A0 and A1. Moreover, the panelists accurately reflected the relationship between the scores for spreadability (smooth texture) and lumpy texture between groups A and B.

According to the overall acceptability scores, A3 (82.5% *HP*, 14.5% *GM*, 1.5% *Stb*, 1.5% *SMP*) had high scores for color, odor and taste, while A2 (83.5% *HP*, 15% *GM*, 1.5% *Stb*, 0% *SMP*) had a more consistent texture. As a result, these two *GMHB* formulations were the most preferred of the options. Similarly, Ardakani et al.^54^ suggested that the best formula for the pistachio nut paste is 72.99–82.99% roasted kernels and 15–25% sugar. In addition, Çapanoğlu and Boyacıoğlu^17^ reported that the addition of *Stb* to almond paste negatively affected the texture, while the samples to which maltose syrup was added were sensually more appreciated, balanced the moisture content, showed a bright appearance and improved the texture. In contrast to these findings, *Stb* improved the homogeneous structure and prevented oil separation (phase separation) in *GMHB* samples in the present study. On the other hand, the effect of *GM* on the sensory properties of *GMHB* was similar to the effect of maltose syrup on *HP*. In other words, the incorporation of *GM* into *HP* resulted in a shiny appearance and improved texture, as well as balancing the moisture content. Moreover, the results of the present study showed that *HP* and *GM* had a stronger effect on taste and odor than *Stb* and balanced the flavor in samples containing *SMP*. Upon evaluating the stability and sensory evaluation results shown in Figure 2a,b together, it is concluded that formula A3 is suitable for future commercial production, as it meets sensory property requirements and does not present any stability issues. While formula A2 is also acceptable based on sensory properties, it does present stability concerns. In this case, it could experiment with different stabilizers and additive ratios, or consider using an emulsifier in addition to the stabilizer.

## 4 Conclusions

This study is one of the rare studies in which the *GMHB* formulations were developed using a natural sugar source (*GM*), their physicochemical and sensory properties were determined, and their shelf life was determined through stability. As *GM* increased in *GMHB*, *TT*, *TPC*, and *HMF* decreased in Group B formulations compared to Group A means, but antioxidant activity, the amount of organic acids from molasses, viscosity, Hunter L*, a*, and b* values increased.

In terms of mineral content, the microelement profile of *GMHB* samples ranked as follows: Fe > Mn > B > Zn > Cu > Al > Sr > Ni > Ba > Cr > Ti > Co > Mo > Se. Due to the higher *HP* content, Group-A samples were richer in microelements when compared to Group-B samples.

The results of the present study indicate that the amounts of *GM*, *HP*, and *Stb*, along with storage temperature (*T*), time (*t*), and particularly the *GM*/*HP* ratio, significantly influence oil separation or emulsion stability. In *GMHB* mixtures, a decrease in the *HP* ratio increased *AOS* and *COS*, while higher storage temperatures affected *AOS* in all samples. It was also found that the presence of *SMP* did not impact *COS* across all mixtures stored at 25 and 45 °C. It was determined that a *GM* ratio of 25% or higher, or an HP ratio of less than 75%, resulted in a robust structure, thick consistency, and darker coloration, which reduced the hazelnut flavor while minimizing oil leakage.

The longest shelf life determined using *COS*, according to 36.6% *US*, was recorded for samples A1, A2, and A3 at 25 °C, and for A1 at 45 °C, as (greater than 250 days). Samples A2 (83.5% *HP*, 15% *GM*, 1.5% Stb, 0% *SMP*) and A3 (82.5% *HP*, 14.5% *GM*, 1.5% Stb, 1.5% *SMP*), which do not obscure the hazelnut aroma and odor and have the most balanced taste with molasses, received the highest sensory acceptability scores, indicating that they can be consumed as suitable alternatives to the control sample.

While commercially produced hazelnut spreads are made with *HP*, *SBRS* or beet sugar, emulsifiers, stabilizers, flavor compounds (vanilla), and fillers (whey or milk powder, etc.), the pastes obtained in this study used only a small amount of *SMP* as fillers, without the addition of emulsifiers, flavor compounds, *SBRS*, artificial sweeteners, or beet sugar. Therefore, *GMHB* is a natural product without many additives. The compatibility of the sugar source, *GM*, with the hazelnut flavor and the nutritional properties of the fruit (grapes) are superior to commercially produced pastes. In conclusion, *GMHB* offers a functional food option that is high in minerals, phenolics, essential fatty acids, vitamins, and dietary fiber, as well as oil and fruit sugars, which is particularly beneficial for individuals with high energy needs.

Furthermore, in this context, the noteworthy research results we have obtained regarding composition properties, product stability, shelf life, and sensory analysis are expected to serve as a valuable resource for researchers and within industrial organizations focused on R&D initiatives on this topic.

In the future, further studies are necessary to develop and optimize new nut-based formulations that align with consumer taste preferences, to explore the use of different stabilizers for stabilization, and to assess microbial quality during storage processes.

## Figures and Tables

**A1 figA1:**
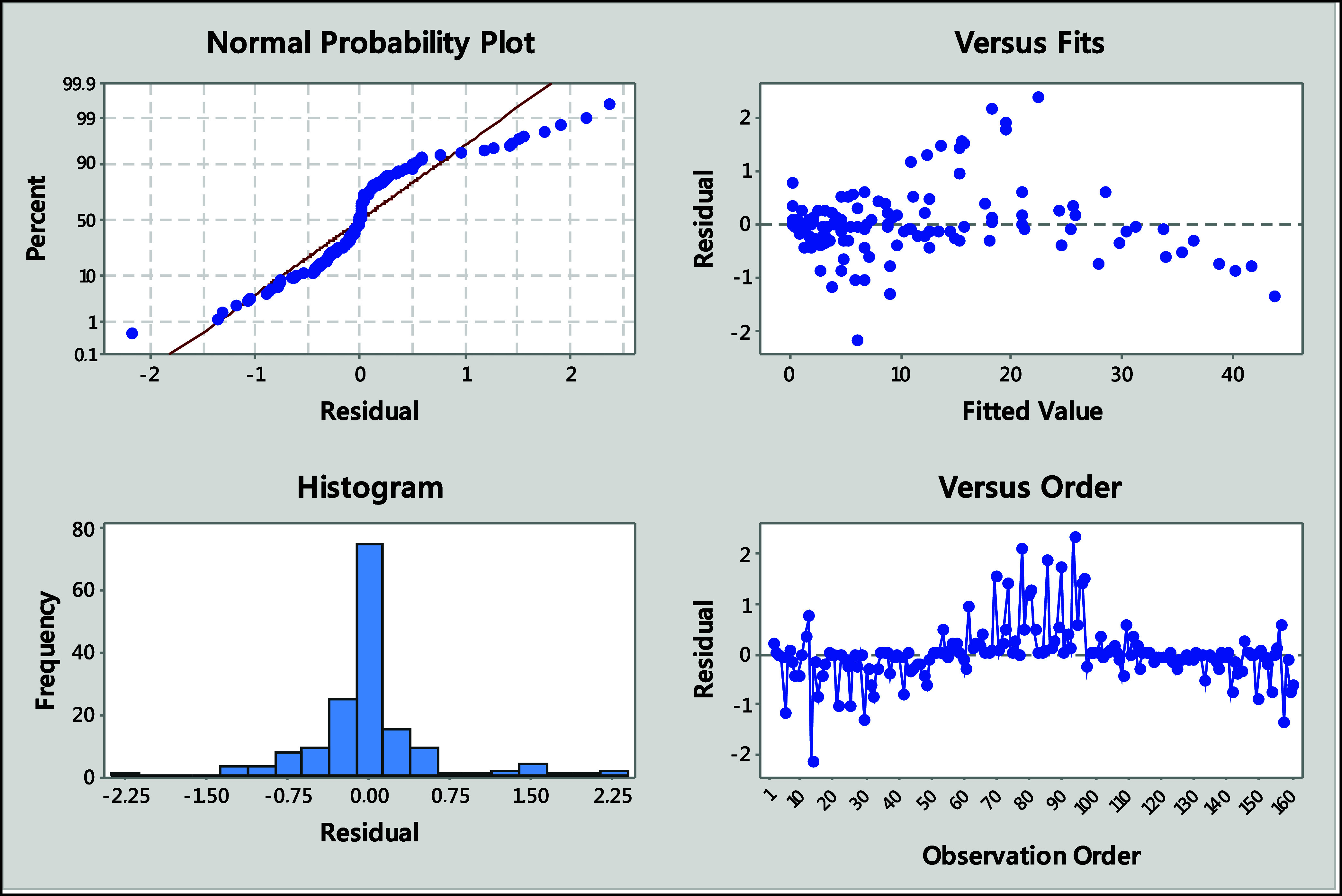
Residual plots for *COS* (%).

## Data Availability

The data supporting this study’s findings are available from the corresponding author upon reasonable request.

## Appendices

### Appendix 1

Table [Table tblA1]

**A1 tblA1:** *ANOVA* Analysis of *GMHB* Formulations for *COS* Value at 25 and 45 °C

source	DF	adj SS	adj MS	*F*-value	*P*-value
time (day)	1	4709.5	4709.49	11050.46	0.000
samples	15	140.9	9.39	22.04	0.000
time (day)*samples	15	3300.8	220.05	516.34	0.000
error	128	54.6	0.43		
total	159	17,108.0			

### Appendix 2

Table [Table tblA2]

**A2 tblA2:** Model Summary

*S*	*R*-sq	*R*-sq (adj)	*R*-sq (pred)
0.652825	99.68%	99.60%	99.50%

### Appendix 3

Table [Table tblA3]

**A3 tblA3:** Coefficients, Standard Errors, *T*-Values, and *P*-Values of the Selected Model for *GMHB* Formulations at 25 and 45 °C

term	coef	SE coef	*T*-value	*P*-value
constant	1.2105	0.0874	13.86	0.000
time (day)	0.45174	0.00430	105.12	0.000
samples				
A0–25	–0.418	0.338	–1.24	0.219
A1–25	–1.211	0.338	–3.58	0.000
A2–25	–1.135	0.338	–3.35	0.001
A3–25	–1.046	0.338	–3.09	0.002
B0–25	2.320	0.338	6.86	0.000
B1–25	–1.199	0.338	–3.54	0.001
B2–25	–0.567	0.338	–1.68	0.096
B3–25	–0.258	0.338	–0.76	0.446
A0–45	1.301	0.338	3.85	0.000
A1–45	–1.143	0.338	–3.38	0.001
A2–45	–1.119	0.338	–3.31	0.001
A3–45	–1.318	0.338	–3.90	0.000
B0–45	4.587	0.338	13.56	0.000
B1–45	–0.539	0.338	–1.59	0.114
B2–45	1.258	0.338	3.72	0.000
B3–45	0.487	0.338	1.44	0.153
time (day)*samples				
A0–25	0.1794	0.0166	10.78	0.000
A1–25	–0.4517	0.0166	–27.14	0.000
A2–25	–0.4491	0.0166	–26.98	0.000
A3–25	–0.4359	0.0166	–26.19	0.000
B0–25	0.5367	0.0166	32.25	0.000
B1–25	–0.3332	0.0166	–20.02	0.000
B2–25	–0.3090	0.0166	–18.56	0.000
B3–25	–0.1690	0.0166	–10.15	0.000
A0–45	0.6011	0.0166	36.12	0.000
A1–45	–0.4468	0.0166	–26.84	0.000
A2–45	0.0323	0.0166	1.94	0.055
A3–45	0.1132	0.0166	6.80	0.000
B0–45	0.5734	0.0166	34.45	0.000
B1–45	–0.0871	0.0166	–5.23	0.000
B2–45	0.2283	0.0166	13.72	0.000
B3–45	0.4173	0.0166	25.07	0.000

### Appendix 4

Figure [Fig figA1]
